# Effect of Laser Quenching on Wire–Powder Collaborative Arc Additive Manufacturing of Ti6Al4V-Cu Alloys with 2.4% and 7.9% Copper Content

**DOI:** 10.3390/ma17246176

**Published:** 2024-12-18

**Authors:** Xingyu Zhang, Weimin Wu, Xiangxiang Zhang, Yanhu Wang

**Affiliations:** 1 Shanghai Waterway Engineering Design and Consulting Co., Ltd., Shanghai 200120, China; 13162955916@126.com (X.Z.); zhangxiangxiang2@ccccltd.cn (X.Z.); 2 China International Science & Technology Cooperation Base for Laser Processing Robotics, College of Mechanical and Electrical Engineering, Wenzhou University, Wenzhou 325035, China; 13353328648@163.com

**Keywords:** laser quenching, additive manufacturing, Ti6Al4V-Cu alloys, micro-specimen tensile test, microhardness, corrosive properties

## Abstract

In this work, Ti6Al4V-Cu alloys with different Cu contents (2.4 and 7.9 wt.%) were fabricated using novel wire–powder synchronous arc additive manufacturing to analyze the effect of laser quenching on Ti6Al4V-Cu alloys. The results show that this method can successfully produce Ti6Al4V-Cu alloys with a uniform composition. As the copper content increased, the alloy transitioned from a Widmanstätten structure to a basketweave structure, and the yield strength and tensile strength of the alloy increased by approximately 35% due to grain refinement and the high volume fraction of Ti_2_Cu with eutectic lamellae. The microhardness of the alloys significantly increased after laser quenching, particularly for those with low copper contents (from 311 HV to 510 HV). Laser quenching also enhanced the corrosion resistance of the alloy in a 3.5% NaCl solution.

## 1. Introduction

Titanium is the fourth most abundant structural metal in the Earth’s crust, with a content of 0.61% [1], surpassing that of common metals such as copper, nickel, tin, lead, and zinc. Titanium and its alloys are widely used in the aerospace industry [2,3], medical equipment [4,5,6], and ocean engineering [7] due to their excellent corrosion resistance, biocompatibility, specific strength, fatigue strength, and high performance. They are often referred to as “space metal”, “biometal”, and “marine metal”.

The addition of new elements to titanium can influence the mechanical properties and microstructure of titanium alloys and contribute to the solidification process to refine the grain structure, homogenize the material organization, and improve the material properties [8,9,10]. In a study by Mantri et al. [11], the addition of 0.5 wt.% B to titanium alloys improved the mechanical hardness and sliding wear properties of the alloy. This improvement was attributed to the compounding effect caused by the fine β grains and α precipitates in the alloys, as well as the in situ formation of TiB precipitates. Xu et al. [12] investigated the effect of hydrogen as a solid solution on the deformation performance of near-α titanium alloys, and the solid solution strengthening effect of hydrogen increased the strength of the alloy. The role of beryllium in the grain refinement in titanium alloys was investigated by Bermingham et al. [10], who compared the effects of Cr, Fe, and beryllium on the grain refinement of CP titanium and observed that a much smaller amount of Be was required to promote the same degree of refinement compared to the addition of Fe or Cr. Copper is an affordable metallic element that typically undergoes a β → α + Ti_2_Cu eutectic transition in titanium-based alloys [13,14]. The mechanical properties of additively fabricated titanium–copper alloys are determined by the amount, density, size, and morphology of these precipitates [15,16,17]. Wang et al. [18] found that the proportion of the Ti_2_Cu phase in the deposition increased with increasing copper content. Under additive manufacturing cycle heat treatment, TC4-5.4Cu martensite decomposed into an α-phase with spherical Ti_2_Cu, and TC4-6.8Cu nanoscale lamellar eutectic Ti_2_Cu grew into micrometer-scale pre-eutectic Ti_2_Cu. Zhang et al. [19] compared Ti-6.5Cu and Ti-3.5Cu alloys and found that the eutectic lamella in Ti-6.5Cu significantly increased the strength but decreased the ductility. They also compared Ti-8.5Cu and Ti-6.5Cu alloys and found that Ti-8.5Cu had higher strength due to the higher volume fraction of eutectic lamella but lower ductility due to pre-eutectic Ti_2_Cu particles. In most of the aforementioned studies, powders or filaments alone were predominantly adopted as raw materials, and a laser was employed as the heat source for the in situ synthesis of titanium alloys. However, arc additive manufacturing technology has several advantages over lasers, including a wider application scope, faster manufacturing speed, and lower cost [20]. We have employed a novel wire–powder synchronous arc additive manufacturing technique. This technology not only circumvents the issue of titanium powder oxidation that is prevalent in metal powder additive manufacturing but also addresses the challenges associated with the use of two different metal wires, whose distinct properties can lead to the formation of cracks and pores in the fabricated alloy. The technique effectively fills the melt pool with wire material while simultaneously introducing trace amounts of powder. In the Ti-Cu system, this technology demonstrates two significant advantages; firstly, it achieves efficient and dense wire additive manufacturing with precise micro-powder delivery to refine grain structure and introduce reinforcing phases. Secondly, it facilitates the bonding between titanium alloy and copper, successfully suppressing segregation during the solidification of the titanium–copper alloy.

Various heat treatments have been employed to enhance the microstructure and mechanical properties of titanium alloys and to improve their application prospects [21,22,23]. Laser quenching is a widely used surface heat treatment technique in modern materials science. It involves a heating rate of up to 104~106 °C/s and a cooling rate of up to 106~108 °C/s. A tiny depth- and temperature-controlled heat-affected zone can be formed by rapidly heating the material surface with a high-power laser beam. This zone undergoes martensitic organization during rapid cooling [24], which enhances the hardness and wear resistance of the material surface. This method is particularly suitable for alloys that require high surface hardness and wear resistance.

Significant variations in the microstructure within alloys, resulting from differing copper contents, exert profound influences on their overall properties. In this study, leveraging the titanium–copper binary phase diagram, we in situ synthesized hypoeutectoid (2.4 wt.%) and near-eutectoid (7.9 wt.%) titanium–copper alloys through a novel arc wire–powder synergistic additive manufacturing technique by adjusting the copper powder feeding parameters. We investigated the impact of various copper additions on the microstructure and mechanical properties of the Ti6Al4V alloy. Subsequently, these titanium–copper alloys with different compositions underwent laser quenching treatment to study the changes in their microstructure and properties before and after quenching. This research addresses the gap in the surface hardening of titanium–copper alloys via laser quenching and provides both theoretical foundations and technical support for the application of titanium–copper alloys in specific fields.

## 2. Materials and Methods

### 2.1. Arc Equipment and Laser Equipment

**Figure 1:**
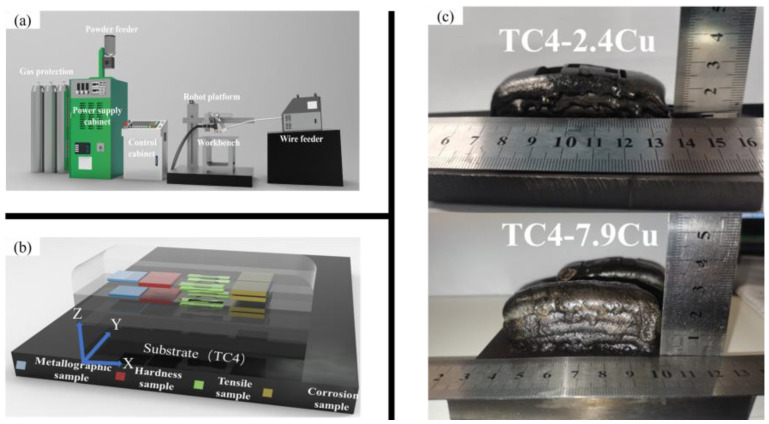
(**a**) Schematic diagram of wire + powder synchronous arc additive manufacturing device; (**b**) schematic diagram of the sampling site; and (**c**) photograph of the deposited sample.

A schematic diagram of the wire + powder synchronous arc additive manufacturing equipment used in this research is shown in Figure 1a. It consists mainly of powder plasma arc additive manufacturing equipment (PPA-AM, DML-VO3AD, Duomu Company, Shanghai, China) with a wire feeder (WPC-600 wire feeder, WEIID Company, Guangzhou, China). Powder plasma arc additive manufacturing equipment mainly consists of three parts, a powder cladding machine, a control system, and a traveling system. The copper powder used in this experiment had a particle size ranging approximately from 40 to 105 μm and a purity greater than 99.9%. It was fed coaxially into the arc gun head via the powder feeder. The Ti6Al4V wire, with a diameter of 1.2 mm, was transported to the molten pool by the side shaft of the WPC-600 wire feeder.

Table 1 shows the composition of the Ti6Al4V wire. The titanium–copper alloy composition was adjusted by controlling the feed of the copper powder and titanium wire. As shown in Table 2, the optimum process parameters were determined after extensive experimentation.

**Figure 2:**
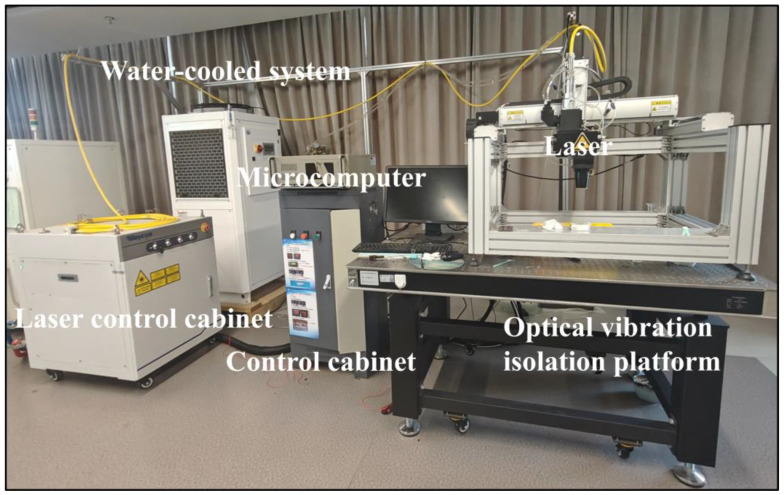
Laser quenching equipment.

A Ti6Al4V sheet with dimensions of 150 × 100 × 10 mm was used as the substrate. Figure 1c shows the deposited sample, which consisted of 5 layers and had a total height of 25 mm. Due to unstable heating, the fusion wire was not used in a timely manner, resulting in poor quality at the ends of the sample. Therefore, for all experiments in this study, only the middle part of the sample was used and two groups of samples were taken from the same location. Electrical discharge machining (EDM) wire cutting was used to cut various patterns along the cross-section of the Ti6Al4V-Cu alloy, including tensile specimens, metallographic observation specimens, and electrochemical corrosion experimental specimens. The specimen location is shown schematically in Figure 1b. This paper defines TC4-2.4Cu as Ti6Al4V with 2.4 wt.% Cu and TC4-7.9Cu as Ti6Al4V with 7.9 wt.% Cu. After heat treatment, the samples are labeled TC4-2.4Cu(L) and TC4-7.9Cu(L). The quenching equipment consisted of a 3 kw RFL-C3000 fiber laser (Shiv Automation, Rajkot, India), Tong Fei cooling equipment (Sanhe, Hebei, China), and a prototype laser processing drive control machine development system (Feicheng, Wuhan, China). Figure 2 shows a physical diagram of the laser quenching equipment. When the quenching temperature reaches 1200 °C, the copper on the surface melts. Upon cooling, the copper redistributed into the alloy in different forms, which affected the alloy. Therefore, after several parameter tests, laser surface quenching of the alloy was carried out at a power of 1050 W and a scanning speed of 6 mm/s.

### 2.2. Observation of Microstructure

The microstructure samples and XRD samples were ground using 80–2000 grit SiC sandpaper until no obvious scratches were visible. Subsequently, they were polished on an MP-2 grinder–polisher (Amazon, Seattle, WA, USA) using a short pile polishing cloth and a 0.25-micron diamond polishing agent until a mirror finish was achieved. After polishing, the samples were ultrasonically cleaned. The phases of the TC4-Cu alloy were detected by XRD (Bruker, Karlsruhe, Germany). The microstructure samples were etched with Kroll’s reagent (10 mL HF, 30 mL HNO_3_, and 130 mL H_2_O) for 5 s and observed by an optical microscope (OM) (OLYMPUS, Tokyo, Japan) and scanning electron microscope (SEM) (JEOL Ltd., Akishima-shi, Japan); the grain size and half-peak widths were analyzed using JADE 6.5 software.

### 2.3. Mechanical Performance Test

The Vickers hardness sample was a rectangular block perpendicular to the deposition direction and was ground to a smooth surface using 60–2000 grit sandpaper. The HV-1000 microhardness tester (HST, Jinan, China) was calibrated using a standard hardness block prior to testing (the equipment was calibrated accordingly for all tests in this study) and after calibration, a load of 9.8 N was applied to the surface of the sample for 15 s to record the microhardness. The surface hardness of the samples with different copper contents was measured before and after quenching. Tensile specimens with a dog bone shape were cut perpendicular to the deposition direction. The specimens had a total length of 13 mm, a scalar segment length of 4 mm, and a cross-section of 1.5 (length) × 0.6 (thickness) mm. Displacement-controlled tensile tests were conducted using a Shimadukin (Kyoto, Japan) AGS-X electronic universal testing machine from Japan at a rate of 0.30 mm/min.

### 2.4. Corrosion Performance Test

**Figure 3:**
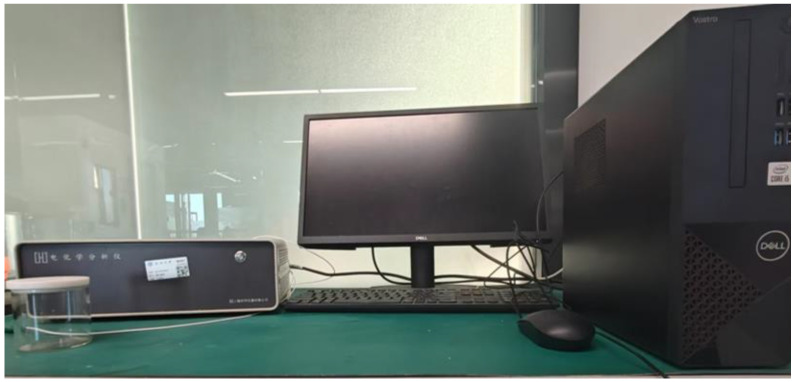
Electrochemical workstation.

The test specimens for electrochemical corrosion were 10 × 10 × 2 mm square blocks that were first ground to a smooth surface using 60–2000 grit sandpaper and then polished to a mirror finish using a silica suspension. To prepare the electrochemical corrosion experimental samples, cold inlaying with acrylic powder and a hardener was used. In this study, the corrosion behavior of TC4-Cu alloys with different copper contents in a 3.5% NaCl solution before and after quenching was investigated using a CHI600E three-electrode electrochemical workstation(Chenhua Company, Shanghai, China), with the sample as the working electrode, a platinum sheet of 10 × 10 × 0.1 mm as the auxiliary electrode, and a saturated calomel electrode as the reference electrode. E_corr_ (corrosion potential) and i_corr_ (corrosion current density) were determined using Tafel curves. The resistance data were analyzed using Zsimpwin 3.60 software. At least three replicates under the same conditions were performed for each set of samples. Figure 3 shows the physical diagram of the electrochemical workstation. Confocal laser scanning microscopy (OLYMPUS, Tokyo, Japan) was then used to observe the surface of the corroded samples and to record the surface roughness, Sa.

## 3. Results and Discussion

### 3.1. Microstructure Observation

**Figure 4:**
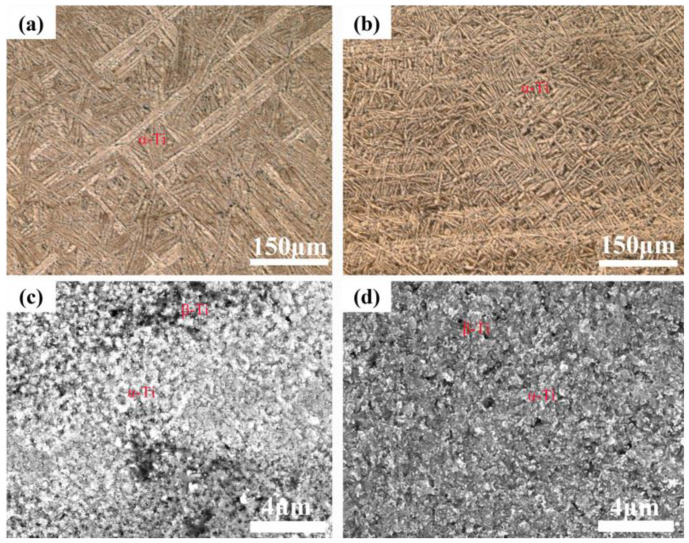
(**a**) Microstructure of deposited TC4-2.4Cu alloy, (**b**) microstructure of deposited TC4-7.9Cu alloy, (**c**) microstructure of deposited TC4-2.4Cu alloy with laser quenching, and (**d**) microstructure of deposited TC4-7.9Cu alloy with laser quenching.

Figure 4 shows the microstructure of the TC4-2.4Cu and TC4-7.9Cu alloys before and after laser quenching, as determined via OM. The OM image of the TC4-2.4Cu alloy shows a typical basket structure composed of original β grains and flaky α-Ti, as shown in Figure 4a. When the copper content increases to 7.9 wt.%, the OM image shows that the microstructure transforms into a fine basket structure, as shown in Figure 4c. After laser quenching, there are obvious α-Ti equiaxed crystals and β-Ti (black), as shown in Figure 4b,c.

A comparison of the TC4-Cu alloys with different Cu contents (Figure 4a,b) shows that the lamellar α-Ti in the microstructure is significantly refined with increasing Cu content, and the lamellar α-Ti phase in the TC4-2.4Cu alloys has a greater aspect ratio (approximately 17:1) and narrower lamellar spacing (approximately 2 µm); additionally, it nucleates at the prior β grain boundaries and the number of nuclei is greater. A complete grain boundary α-Ti phase is formed at the prior β grain boundary contact, and as the phase transformation proceeds, the α-Ti phase grows along a certain orientation toward the intercrystalline region to form a cluster. In contrast, the α-Ti lamellae in the Ti-7.9Cu alloys have a lower aspect ratio (approximately 7:1), as well as a larger lamellar spacing (about 8 µm), and they nucleate at the prior β grain boundaries and within the grains. The growth direction of the lamellar α-Ti phase inside the grains is influenced by the cooling conditions, resulting in the interlacement of the lamellar α-Ti phases and the formation of lattice basket features.

Due to the rapid cooling effect of laser quenching, according to the interdependence theory, laser quenching can form a small nucleation critical undercooling ΔTn, which in turn generates a larger compositional undercooling ΔTCS. This promotes an increase in nucleation and the formation of small, evenly distributed effective nucleation particles. The average spacing between these nucleation particles is small, effectively limiting the grain growth space and achieving the effect of grain refinement [20]. Additionally, as the copper content increases, the influence of the growth restriction factor (Q) becomes more pronounced, further inhibiting grain growth [25]. This restrictive effect suppresses the growth of acicular α-Ti, while equiaxed crystals, due to their lower surface energy and higher stability, gradually gain an advantage in the competitive growth process, ultimately achieving the transformation from acicular α-Ti to equiaxed crystals (as shown in Figure 4b,d).

**Figure 5:**
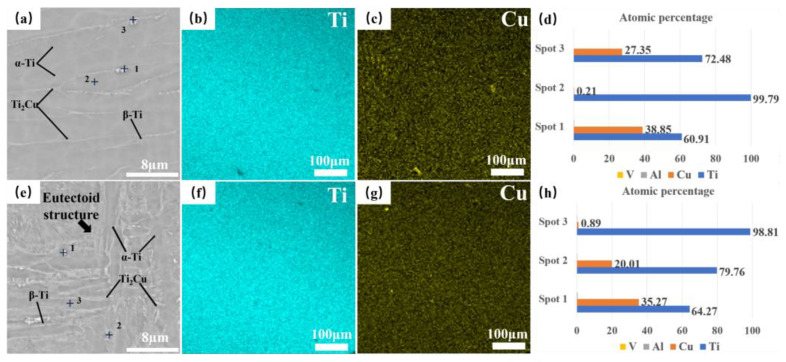
(**a**) The microstructure and characteristic phase structure of the deposited TC4-2.4Cu alloy, (**b**–**d**) Ti and Cu distribution maps of the TC4-2.4Cu alloy, (**e**) the microstructure and characteristic phase structure of the deposited TC4-7.9Cu alloy, and (**f**–**h**) Ti and Cu element distribution map of the TC4-7.9Cu alloy.

**Figure 6:**
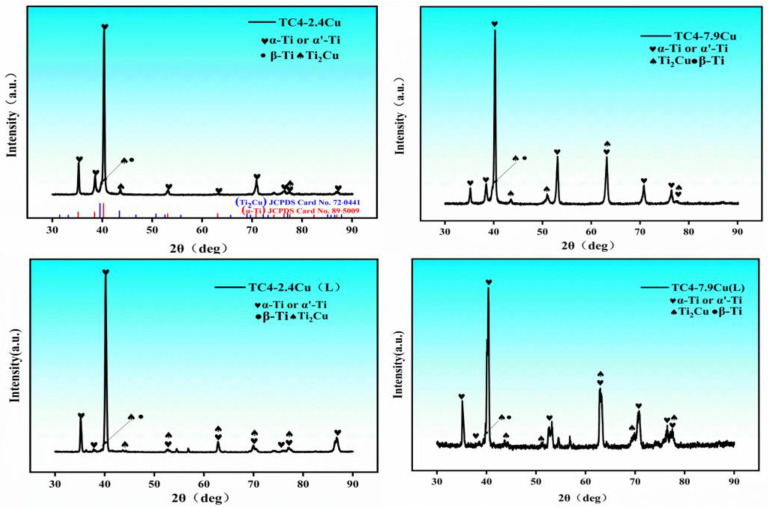
X-ray diffraction of each group of samples.

Figure 5a shows the SEM image of the TC4-2.4Cu alloy, which shows that the microstructure of the alloy is mainly composed of a lamellar α-Ti phase with a tiny rod-like phase. In this case, a small rod-like phase is formed at the grain boundaries of the lamellar structure when the remaining β-Ti undergoes eutectic transformation as the alloy temperature decreases to the eutectic transformation temperature [26]. A few spherical phases were observed inside the lamellar α-Ti phase grains. This peak is initially attributed to the Ti_2_Cu phase, considering that the solubility of Cu in the α-Ti phase decreases as the alloy cools to room temperature and that its precipitation location is inside the α-phase. This was confirmed by the XRD and EDS results. The tiny rod-like and spherical phases observed in the microstructure of TC4-2.4Cu are both Ti_2_Cu phases with an atomic ratio close to 2:1. The EDS results are shown in Figure 5. Figure 5b,c,f,g shows the distribution of elements in the alloy, with no significant segregation observed. This indicates that the alloy composition prepared by this method is relatively uniform. The XRD results are shown in Figure 6. An EDS analysis of the precipitated phase in the TC4-7.9Cu sample showed that the precipitated phase was the Ti_2_Cu phase, with the difference that the Ti_2_Cu phase had different morphologies and contents with increasing Cu content in the TC4-Cu alloy. In the TC4-7.9Cu alloy, the Ti_2_Cu phase in the microstructure is lath-like and massive, and traces of the spherical Ti_2_Cu phase are also observed in the lamellar α-Ti phase, as shown in Figure 5e. The bulk phase is probably the pre-eutectic Ti_2_Cu phase.

The XRD results are shown in Figure 6. In the TC4-2.4Cu and TC4-7.9Cu alloys, the microstructure was mainly composed of the α-Ti phase (α’-Ti phase), β-Ti phase, and Ti_2_Cu phase. One may observe that the amount of Ti_2_Cu phase in the alloy increases with increasing copper content. Using JADE 6.5 software, the Ti_2_Cu phase content was calculated to be 4.7 wt.% for the TC4-2.4 Cu alloy and 8.9 wt.% for the TC4-7.9 Cu alloy, an increase of 4.2 wt.%. This result is consistent with the phenomenon of increased Ti_2_Cu phase content under scanning electron microscopy. In addition, the α-Ti phase diffraction peaks show a left-shifted trend. The reason for this phenomenon may be that the lattice parameters of the α-phase in TC4-2.4Cu are a = 2.944 nm and c = 4.678 nm, the lattice parameters of the Ti_2_Cu phase are a = 2. 944 nm and c = 10.786 nm, those of the α phase in TC4-7.9Cu are a = 2.951 nm and c = 4.684 nm, and the lattice parameters of the Ti_2_Cu phase are a = 2.944 nm and c = 10.786 nm. Cu atoms replaced the Ti atoms in the α-Ti cell to form a substitutional solid solution, thus distorting the α-Ti crystal lattice array and increasing the lattice parameter. The Bragg equation (nλ = 2dsin(θ)), which relates the diffraction order (n), the wavelength of the incident light (λ), the crystal plane spacing (d), and the diffraction angle (θ), shows that the diffraction angle and the crystal plane spacing have an inverse relationship when the wavelength of the incident light and the diffraction order remain constant. When the copper content increases, the diffraction angle θ of the α-Ti phase diffraction peak decreases, shifting toward the low-angle direction. After laser quenching at 1200 degrees Celsius, the Ti_2_Cu content in TC4-2.4Cu was elevated to 10.0 wt.% compared to 9.6 wt.% in the TC4-7.9Cu alloy.

**Figure 7:**
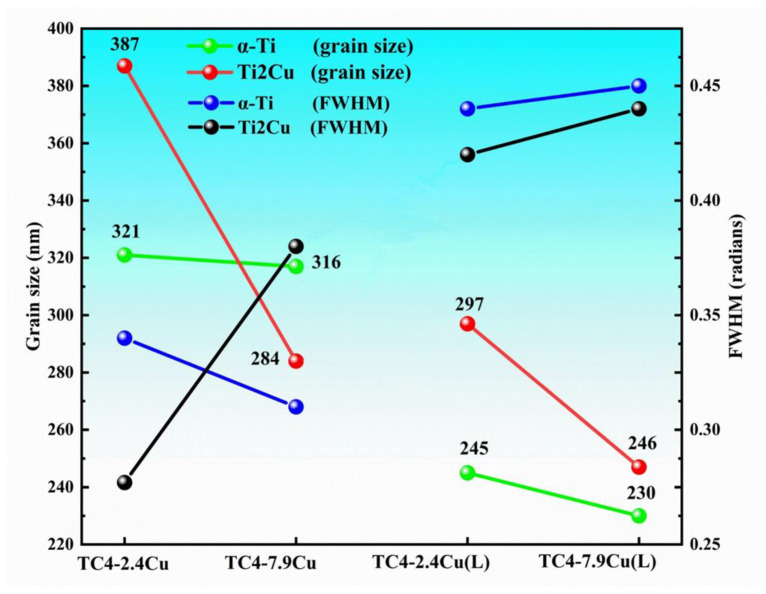
Average grain size and half-peak width for each group of samples.

Figure 7 shows the average values of the grain size and half-peak width calculated from each set of diffraction peaks of the α-Ti phase and Ti_2_Cu phase. The results indicate an inverse relationship between the amount of copper and the grain size of the alloy. The average grain size of TC4-2.4Cu alloy after laser quenching is 240 nm, which is 25% less than that before quenching. The average grain size of the TC4-7.9Cu alloy after laser quenching is 230 nm, which is 27% less than that before quenching. Laser quenching can further refine the grain size. With the rapid cooling effect of laser quenching, with increasing copper content, the subcooling range of the front edge of the solid/liquid interface increases and the degree of grain refinement increases.

### 3.2. Hardness Analysis

**Figure 8:**
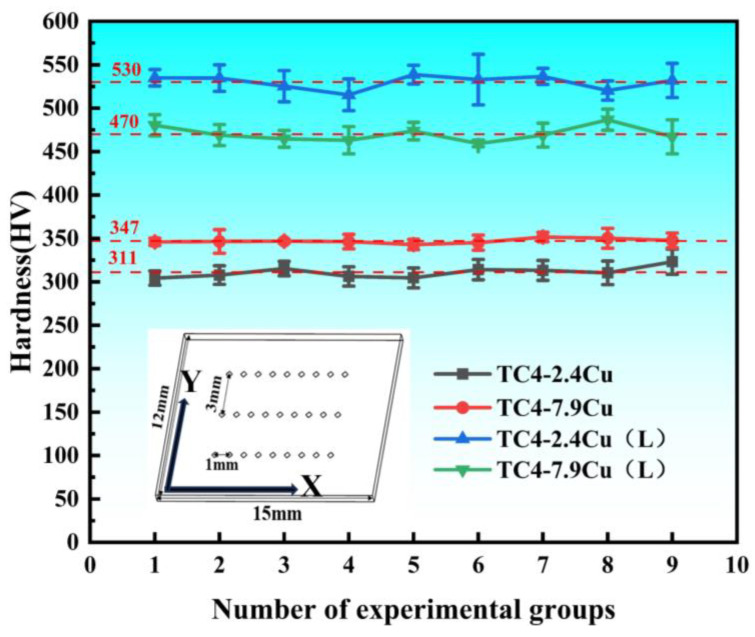
Hardness test results.

A rectangular microhardness sample, 15 mm long and 12 mm wide, was obtained perpendicular to the direction of deposition, and a total of 27 hardness value points were taken from its surface, with a generally rectangular arrangement of the dot matrix. Measurements were taken at 1 mm intervals in the X-direction and repeated nine times, with two additional sets taken at 3 mm intervals in the Y-direction using the same method. Figure 8 displays the results. The TC4-7.9Cu alloy has a hardness of approximately 347 HV, which is approximately 11.6% greater than that of TC4-2.4Cu (311 HV) and slightly greater than that of the commercial TC4 alloy (300 HV). The hardness enhancement is associated with smaller grains and the generation of more Ti_2_Cu. After laser quenching, the microhardness of the alloys significantly improved, particularly for the TC4-2.4Cu alloy with a lower copper content, where a higher thermal conductivity and faster cooling rate resulted in an average hardness of 530 HV and an increase of 70.4%. The hardness increase rate of the TC4-7.9Cu alloy with a higher Cu content also reached 35.4% (470 HV). Grain refinement, solution strengthening, and precipitation strengthening are the main reasons for the significant improvement in the microhardness of the alloy after quenching. The strength of the TC4-2.4Cu alloy increases after laser quenching because under high-speed cooling, copper atoms cannot precipitate and are solidly dissolved in the titanium atomic lattice, and solid solution strengthening plays a major role.

### 3.3. Tensile Test Analysis

**Figure 9:**
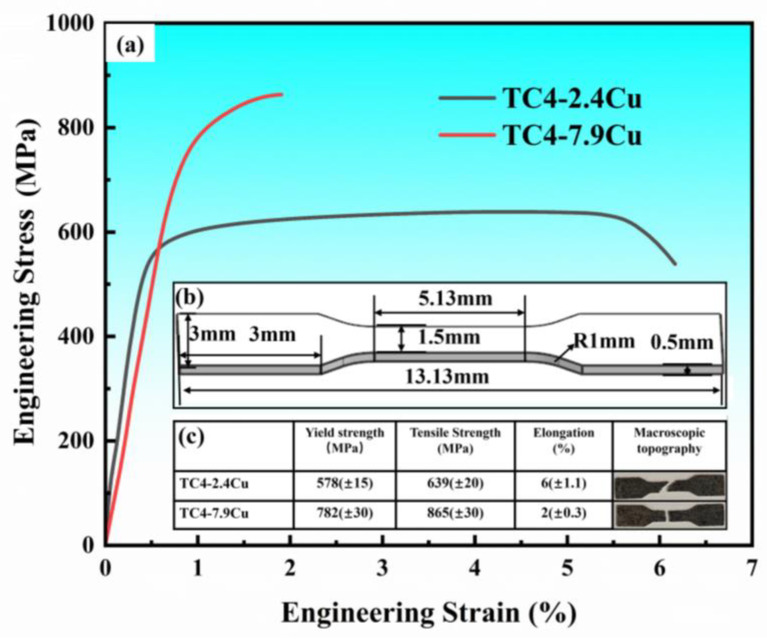
(**a**) Engineering stress–strain curves, (**b**) The dimensional diagram of tensile test specimen, (**c**) Specific results of tensile test.

The elastic properties of the specimens were investigated, and the results are shown in Figure 9. Figure 9a shows the tensile stress–strain curves of the TC4-Cu alloys with different compositions. Figure 9b shows the dimensional drawings of the tensile specimens, and each group of tensile tests was repeated three times under the same conditions to ensure the accuracy of the experimental results. Figure 9c shows the specific values of the yield strength, ultimate tensile strength, and elongation for the two groups of composition alloys. As the copper content increases, the plasticity of the alloy decreases and the strength increases. Compared with those of the TC4-2.4Cu alloy, the yield strength and tensile strength of the TC4-7.9Cu alloy increases by approximately 35%, while the elongation is reduced by 66%.

**Figure 10:**
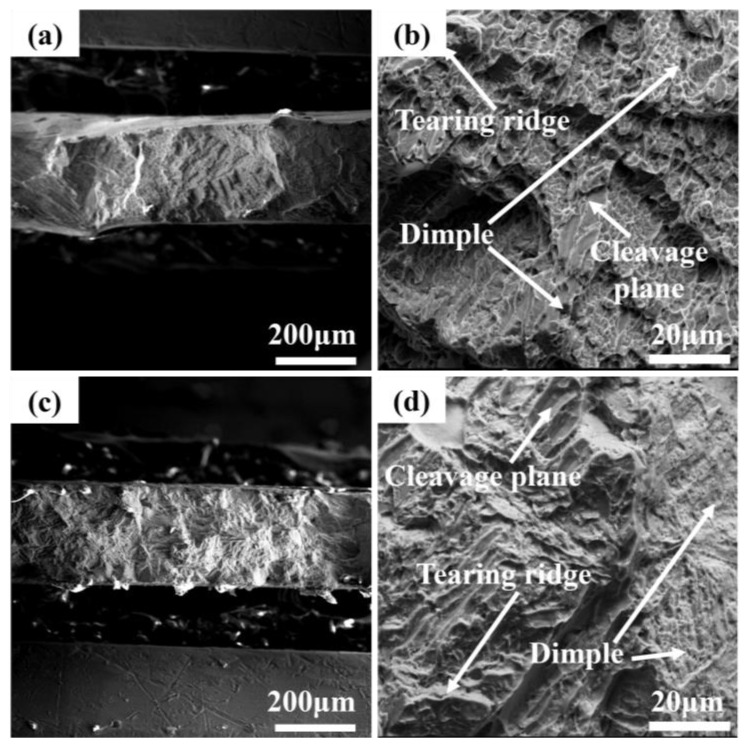
Tensile fracture morphology. (**a**,**b**) TC4-2.4Cu, (**c**,**d**) TC4-7.9Cu.

Figure 10 shows the micro-morphology of the tensile fracture of the TC4-Cu alloys with different compositions. Figure 10 illustrates that both groups of TC4-Cu alloys with different compositions exhibit a combination of dimples, tearing ridges, and cleavage platforms during tensile fracture, indicating a mixed tough and brittle fracture mechanism. However, there are differences in the morphology of the TC4-7.9Cu alloy tensile fracture (Figure 10c,d) compared to that of the TC4-2.4Cu alloy (Figure 10a,b). The fracture of the TC4-7.9Cu alloy exhibits a greater number and area of cleavage platforms, while the number and depth of dimples are smaller. This could be attributed to the brittle behavior of Ti_2_Cu intermetallic compounds.

The fracture morphology results are in concordance with the data obtained from the tensile experiments. Combined with the microstructure of the alloy, the fine Ti_2_Cu phases in the TC4-2.4Cu alloy can act as barriers to dislocation movement. When dislocations encounter these particles, they change paths, making it more difficult for them to move, absorbing more plastic deformation energy and improving the plasticity of the alloy. The TC4-7.9Cu alloy has improved strength due to its high Ti_2_Cu content, but its ductility is reduced because the larger Ti_2_Cu phase creates a stress concentration area that is prone to crack formation when external stresses are applied.

### 3.4. Corrosion Behavior

**Figure 11:**
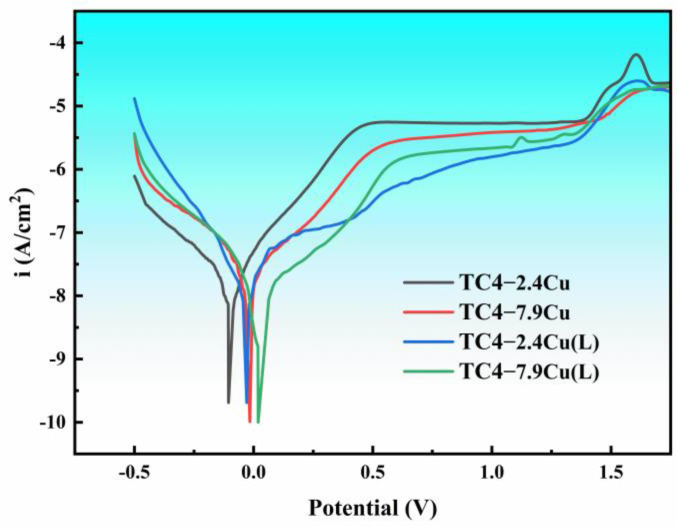
The potentiodynamic polarization curves.

The time required for the alloy to reach a stable state in different electrolyte solutions varies. In this study, the system was tested for 1 h at open circuit voltage to ensure that the system reached a stable state, and the electrochemical impedance spectra were obtained by applying a perturbation voltage of 5 mV to the material at a frequency of 10^5^ Hz to 10^−2^ Hz at an open circuit voltage. The voltage range used for the Tafel curve test was -500 mV to 2000 mV with a scan rate of 0.5 mV/s. After the corrosion test, no corrosion pits were visible on the alloy surface. Analysis of the kinetic potential polarization curve indicated that the TC4-Cu alloy exhibited clear passivation characteristics in a 3.5% NaCl solution, with a wider passivation interval (Figure 11). This suggests that the alloys produced using wire–powder synchronous arc additive manufacturing technology have excellent corrosion resistance properties.

Table 3 displays the electrochemical parameters, including the corrosion potential (E_corr_) and corrosion current density (i_corr_). The E_corr_ values of the TC4-Cu samples from both compositions showed higher potential values and lower corrosion current values than those of commercial TC4, indicating that the addition of copper can promote the formation of a more compact and stable passivation film on the alloy surface, thereby improving the corrosion resistance of the alloy. Despite containing more Ti_2_Cu phases, the XRD results show that the TC4-7.9Cu alloy has a higher corrosion potential and lower corrosion current density, which means that its corrosion resistance is better than that of TC4-2.4Cu. The improved performance of the alloy can be attributed to the uniform distribution of the slatted Ti_2_Cu phases and the reduced spacing between them. This distribution pattern provides an effective “protective layer” [27] for the alloy, which significantly reduces the potential for galvanic coupling corrosion and enhances the corrosion resistance of the alloy by changing the cathode-to-anode ratio between the Ti_2_Cu phase and the α-Ti phase. Laser quenching further enhances the corrosion resistance of two-component alloys by improving the densification of the passivation film.

**Figure 12:**
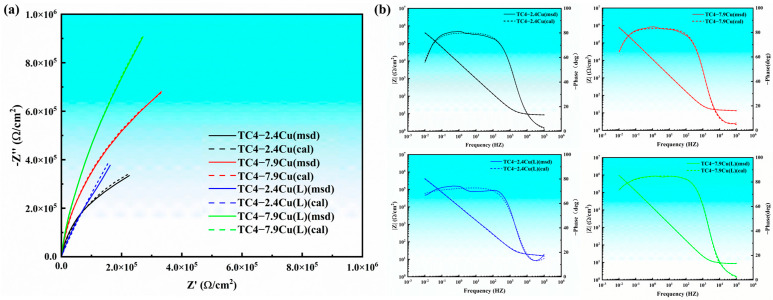
(**a**) Nyquist plot diagram; (**b**) Bode plot diagram and Bode phase.

To further analyze the corrosion resistance of the alloy, electrochemical impedance spectroscopy (EIS) was performed. The corresponding characterization curves are shown in Figure 12a. Sample TC4-7.9Cu(L) exhibited the largest impedance arc, while sample TC4-2.4Cu exhibited the smallest. The smaller the radius of the arc is, the more susceptible the alloy is to corrosion reactions. Additionally, an increase in the copper content enhances the corrosion resistance of the alloy, and the laser quenching treatment further improves its corrosion resistance. The Bode diagram is shown in Figure 12b. In the high-frequency range of 10^4^–10^5^ Hz, the modulus remains constant, indicating a phase angle of 0°. The impedance values in this range are representative of the solution resistance (Rs). The maximum phase angle occurs in the mid-frequency range (10^3^–10^−1^ Hz), indicating a two-layer structure of the passivation film with two time constants. When a titanium alloy corrodes, the passivation film formed on its surface should consist of an inner barrier layer and an outer hydroxide layer. The mode corresponding to the low-frequency range represents the level of corrosion resistance of the alloy. The results illustrate that the TC4-7.9Cu(L) sample exhibits the highest modulus, while the TC4-2.4Cu sample exhibits the lowest modulus.

**Figure 13:**
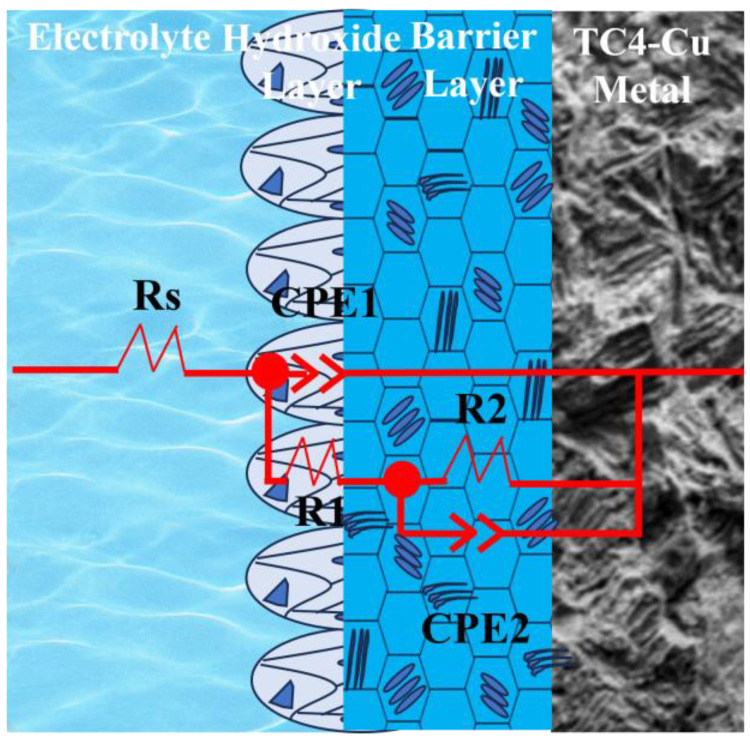
Electrochemical equivalent circuit diagram.

ZsimpleWin 3.60 software was used to fit the data based on the above characteristics (the fitting results are indicated by dashed lines in Figure 12), and the equivalent circuit model used was Rs (Q_1_(R_1_(Q_2_R_2_)), as shown in Figure 13. In this equation, Rs represents the solution resistance, while R_1_ and R_2_ represent the charge transfer resistances of the hydroxide and barrier layers, respectively. Additionally, CPE_1_ and CPE_2_ represent the impedances of the hydroxide and barrier layers, respectively, which are integral components of the bilayer passivation film. The fitting results were verified using Zview 2.70 software and showed an error of less than 10%, indicating that the test results are reliable. The EIS equivalent circuit parameters for each sample are shown in Table 4. The fitting error rates of each component are indicated in parentheses in Table 4.

**Figure 14:**
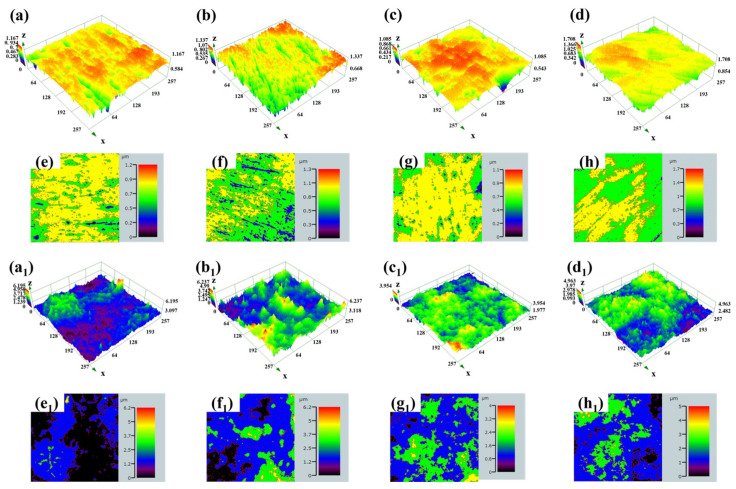
(**a**–**h**) 3D morphology before corrosion; (**a_1_**–**h_1_**) 3D morphology after corrosion.

To more intuitively observe the surface morphology after corrosion, confocal laser scanning microscopy was used to examine the corroded surface, and the results are shown in Figure 14. After measurement, the surface roughness Sa of the TC4-2.4Cu alloy was 0.053 (±0.006) μm before corrosion (Figure 14a,e), and after corrosion, the Sa value reached 0.091 (±0.004) μm (Figure 14b,f), with a change of 72%. The surface roughness of the TC4-7.9Cu alloy changed from 0.047 (±0.003) μm (Figure 14c,g) to 0.061 (±0.004) μm (Figure 14d,h), a change of 30%. Moreover, the corrosion resistance of the TC4-7.9Cu alloy is greater than that of the TC4-2.4Cu alloy. After the laser quenching treatment, the surface roughness Sa values of the alloys improved. The Sa value of the TC4-2.4Cu(L) alloy was 0.230 (±0.007) μm (Figure 14a_1_,e_1_), and the Sa value of this alloy after the electrochemical corrosion test was 0.328 (±0.007) μm (Figure 14b_1_,f_1_), with a change of 43%. The Sa value of the TC4-7.9Cu(L) alloy is 0.225 (±0.001) μm (Figure 14c_1_,g_1_), and after corrosion, the Sa value became 0.258 (±0.004) μm (Figure 14d_1_,h_1_), with a change of approximately 15%.

## 4. Conclusions

In this work, TC4-2.4Cu and TC4-7.9Cu alloys were prepared using a wire + powder synchronous arc additive manufacturing method. The two alloys with different copper alloying additions underwent laser surface hardening, and their microstructures, mechanical properties, and corrosion properties were compared. The results showed the following:

(1) The wire + powder synchronous arc additive manufacturing method can effectively achieve the simultaneous feeding of silk and powder. Alloys produced by this method have a smooth shape, better molding quality, and a more homogeneous composition.
(2) As the copper content increases, that is, compared with those of the TC4-2.4Cu alloy and TC4-7.9Cu alloy, the structure of the alloy changes from the Widmanstädter structure to the alloy basket structure, accompanied by the Ti_2_Cu phase. An increase in hardness from 6.6% to 8.2% and a shift in α-Ti phase diffraction peak to the left resulted in an 11.6% increase in hardness, an increase in the alloy yield strength and ultimate tensile strength of approximately 35%, and an increase in the corrosion resistance.
(3) Laser quenching increases the surface roughness of the alloy, but after quenching, the grain size is refined, resulting in an increase in the hardness of the alloy by 35.4% and 70.4%, respectively, simultaneously enhancing its corrosion resistance.

## Figures and Tables

**Table 1:** Ti6Al4V wire composition (wt.%).

**Element**	Ti	Fe	C	N	H	O	Al	V
**Content**	Rem	0.20	0.07	0.02	0.01	0.12	5.9	4.0

**Table 2:** Parameters of the additive manufacturing process.

	TC4-2.4Cu		TC4-7.9Cu	
Parameter	Value	Unit	Value	Unit
Deposition current	105	A	105	A
TC4 wire speed	9.8	g/min	9.8	g/min
Copper powder speed	0.74	g/min	1.5	g/min
Travel speed	400	mm/min	400	mm/min
Shielding Ar gas	12	L/min	12	L/min
Powder feeding Ar gas	3	L/min	3	L/min
Dwell time between deposition layers	4	min	4	min
The angle between the torch and filler wire	60	°	60	°
Distance between the torch and workpiece	15	mm	15	mm

**Table 3:** Electrochemical data of the four samples.

Parameter	TC4-2.4Cu	TC4-7.9Cu	TC4-2.4Cu(L)	TC4-7.9Cu(L)	Commercial TC4 Alloy
E_corr_ (V)	−0.08 (±0.025)	−0.012 (±0.071)	−0.014 (±0.063)	0.007 (±0.011)	−0.6468
i_corr_ (μA/cm^2^)	0.035 (±0.011)	0.019 (±0.008)	0.014 (±0.03)	0.007 (±0.014)	0.26

**Table 4:** The simulated equivalent circuit parameters.

Parameters	TC4-2.4Cu	TC4-7.9Cu	TC4-2.4Cu(L)	TC4-7.9Cu(L)
Rs (Ω)	8.51 (0.74%)	12.40 (2.77%)	5.68 (9.77%)	2.66 (0.40%)
CPE_1_ (S-sec^n)	1.83 × 10^−5^ (4.07%)	2.35 × 10^−6^ (9.84%)	5.50 × 10^−6^ (5.80%)	9.51 × 10^−6^ (0.45%)
R_1_ (Ω)	13.70 (9.77%)	2.80 (4.94%)	13.58 (5.61%)	18.42 (6.24%)
n_1_, hydroxide layer	0.89 (5.01%)	0.94 (0.49%)	0.69 (8.08%)	0.66 (0.08%)
CPE_2_ (S-sec^n)	6.531 × 10^−6^ (8.57%)	1.317 × 10^−5^ (1.84%)	1.572 × 10^−5^ (1.30%)	6.708 × 10^−6^ (5.45%)
R_2_ (kΩ)	1009 (2.82%)	2222 (3.28%)	11,940 (9.95%)	442,400 (9.36%)
n_2_, hydroxide layer	0.89 (8.93%)	0.93 (3.21%)	0.84 (1.30%)	0.89 (2.26%)
Chi-squared (x^2^)	4.076 × 10^−4^	3.337 × 10^−4^	2.047 × 10^−3^	2.14 × 10^−4^

## Data Availability

The original contributions presented in this study are included in the article; further inquiries can be directed to the corresponding author.
